# A predictive model for the severity of COVID-19 in elderly patients

**DOI:** 10.18632/aging.103980

**Published:** 2020-11-10

**Authors:** Furong Zeng, Guangtong Deng, Yanhui Cui, Yan Zhang, Minhui Dai, Lingli Chen, Duoduo Han, Wen Li, Kehua Guo, Xiang Chen, Minxue Shen, Pinhua Pan

**Affiliations:** 1 Department of Dermatology, Xiangya Hospital, Central South University, Changsha, China; 2 National Clinical Research Center for Geriatric Disorders, Changsha, China; 3 Hunan Engineering Research Center of Skin Health and Disease, Changsha, China; 4 Hunan Key Laboratory of Skin Cancer and Psoriasis, Changsha, China; 5 Department of Respiratory Medicine, Xiangya Hospital, Central South University, Changsha, China; 6 School of Computer Science and Engineering, Central South University, Changsha, China; 7 Department of Social Medicine and Health Management, Xiangya School of Public Health, Central South University, Changsha, China

**Keywords:** COVID-19, elderly patients, severity, nomogram

## Abstract

Elderly patients with coronavirus disease 2019 (COVID-19) are more likely to develop severe or critical pneumonia, with a high fatality rate. To date, there is no model to predict the severity of COVID-19 in elderly patients. In this study, patients who maintained a non-severe condition and patients who progressed to severe or critical COVID-19 during hospitalization were assigned to the non-severe and severe groups, respectively. Based on the admission data of these two groups in the training cohort, albumin (odds ratio [OR] = 0.871, 95% confidence interval [CI]: 0.809 - 0.937, P < 0.001), d-dimer (OR = 1.289, 95% CI: 1.042 - 1.594, P = 0.019) and onset to hospitalization time (OR = 0.935, 95% CI: 0.895 - 0.977, P = 0.003) were identified as significant predictors for the severity of COVID-19 in elderly patients. By combining these predictors, an effective risk nomogram was established for accurate individualized assessment of the severity of COVID-19 in elderly patients. The concordance index of the nomogram was 0.800 in the training cohort and 0.774 in the validation cohort. The calibration curve demonstrated excellent consistency between the prediction of our nomogram and the observed curve. Decision curve analysis further showed that our nomogram conferred significantly high clinical net benefit. Collectively, our nomogram will facilitate early appropriate supportive care and better use of medical resources and finally reduce the poor outcomes of elderly COVID-19 patients.

## INTRODUCTION

Severe acute respiratory syndrome coronavirus 2 (SARS-CoV-2), the causal agent of the coronavirus disease 2019 (COVID-19) pandemic, has posed a considerable threat to global public health [1, 2]. SARS-CoV-2 has been identified as a novel single-stranded ribonucleic acid (RNA) betacoronavirus that shares great phylogenetic similarity with severe acute respiratory syndrome coronavirus [3, 4]. As of April 27, 2020, a total of 2,878,196 confirmed cases were reported, including 198,668 deaths worldwide [5]. Numerous studies have demonstrated that most COVID-19 patients had no symptoms or had mild pneumonia, while a certain proportion of patients, especially elderly patients, were more prone to contracting severe or critical pneumonia or even dying [6–8]. Recent studies showed that patients with severe COVID-19 had a fatality rate 20 times higher than those with non-severe COVID-19 [9, 10]. Therefore, it is important to build a predictive model for the severity of COVID-19 on admission.

Elderly patients are the high-risk group for severe COVID-19 [6]. A study conducted by Liu and his team found that the proportion of COVID-19 patients with grade IV and V pneumonia based on the Pneumonia Severity Index was higher among elderly patients than among young and middle-aged patients [11]. Moreover, Lian et al. conducted a cohort study including 652 younger patients and 136 older patients, and the results suggested that older patients had higher rates of severe COVID-19 and intensive care unit (ICU) admission than younger patients [12]. Furthermore, based on a recent report published by the Chinese Center for Disease Control and Prevention that enrolled approximately 44,500 confirmed cases, patients over 60 years in age accounted for approximately 81% of the total mortality in a nationwide analysis conducted in China [13], which was consistent with data from the United States [14] and Italy [15]. Considering that there is currently no specific medication for COVID-19 [16], early identification of elderly COVID-19 patients at high risk of exacerbation to severe or critical pneumonia is imperative to facilitate appropriate supportive care and reduce poor outcomes.

However, to the best of our knowledge, no risk models have been developed to predict the severity of COVID-19 in elderly patients. In this study, using logistic regression with least absolute shrinkage and selection operator (LASSO) regularization, we found that albumin (ALB), d-dimer and onset to hospitalization (OH) time were significant predictors for the severity of COVID-19 in elderly patients. Based on these factors, we developed an effective risk nomogram with high sensitivity and specificity for accurate individualized assessment of the severity of COVID-19 in elderly patients. Our nomogram will facilitate early appropriate supportive care and better use of medical resources and will finally reduce the poor outcomes of elderly COVID-19 patients.

## RESULTS

### Clinicopathologic characteristics of enrolled elderly patients with COVID-19

**Figure 1:**
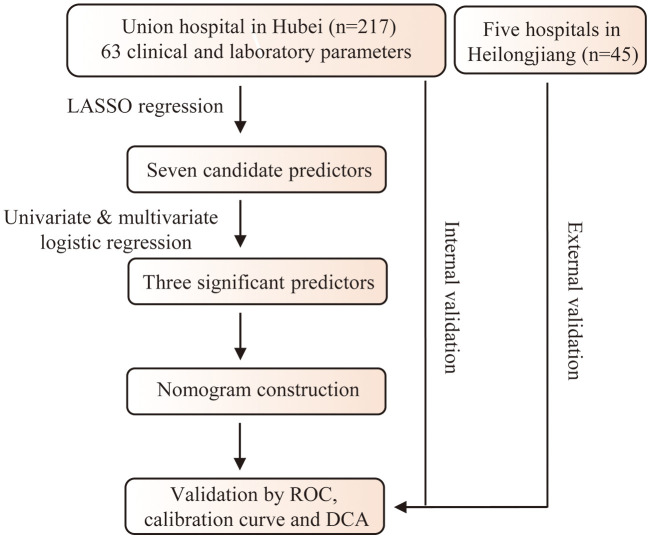
**Flowchart of the study.**

The flowchart of the study is presented in Figure 1. A total of 262 elderly COVID-19 patients were enrolled from six different hospitals in Hubei and Heilongjiang provinces in the study (Table 1). A total of 217 patients in Hubei Province were grouped as the training cohort, and 45 patients in Heilongjiang Province were grouped as the validation cohort. All the patients during hospitalization were followed until discharge from the hospital. Patients who maintained a non-severe condition (recovery or mild or moderate COVID-19) and patients who progressed to severe or critical COVID-19 were assigned to the non-severe and severe groups, respectively. The proportion of severe patients and patients with coronary heart disease was higher in the training cohort. There were no significant differences in age, gender, or other comorbidities between these two cohorts. In the training cohort, patients in the severe group were older than those in the non-severe group (68.0 years, interquartile range (IQR) [64.0 - 75.0] vs. 65.0 years, IQR [62.0 - 70.0], P = 0.002) (Table 2). Compared with patients in the non-severe group, those in the severe group had less OH time (10.0 days, IQR [7.0 - 15.0] vs. 14.5 days, IQR [7.0 - 25.8], P = 0.018). There were no differences in other symptoms or signs between these two groups. Significant differences were observed in the laboratory indicators between these two groups. In summary, patients in the severe group had higher inflammation, a hypercoagulable state, and increased hepatic and renal injury compared with those in the non-severe group.

**Table 1:** Baseline characteristics of the study cohort.

**Variables**	**Training cohort (n=217)**	**Validation cohort (n=45)**	**p-value**
Age (year)	67.0 (64.0-73.0)	66.0 (63.5-76.0)	0.952
Gender, n (%)			
Female	101 (46.5)	21 (46.7)	0.988
Male	116 (53.5)	24 (53.3)	
Severity, n (%)			
Non-severe	52 (24.0)	21 (46.7)	0.002
Severe	165 (76.0)	24 (53.3)	
Comorbidities, n (%)			
Tumor	16 (7.4)	7 (15.6)	0.078
Diabetes	51 (23.5)	9 (20.0)	0.611
Hypertension	87 (40.1)	19 (42.2)	0.791
Coronary heart disease	25 (11.5)	11 (24.4)	0.022
Chronic kidney disease	10 (4.6)	2 (4.4)	1.000*
Chronic respiratory disease	18 (8.3)	2 (4.4)	0.542*

* Calculated with Fisher’s exact test.

**Table 2:** Demographics and characteristics of elderly COVID-19 patients in training cohort.

**Variables**	**Non-severe (n=52)**	**Severe (n=165)**	a**p-value**
Age (year)	65.0 (62.0-70.0)	68.0 (64.0-75.0)	0.002
Gender			
Female	30 (57.7)	71 (43.0)	0.065
Male	22 (42.3)	94 (57.0)	
Comorbidities, n (%)			
Tumor	5 (9.6)	11 (6.7)	0.478
Diabetes	11 (21.2)	40 (24.2)	0.647
Hypertension	16 (30.8)	71 (43.0)	0.116
Coronary heart disease	5 (9.6)	20 (12.1)	0.622
Chronic kidney disease	2 (3.8)	8 (4.8)	1.000*
Chronic respiratory disease	2 (3.8)	16 (9.7)	0.253*
OH time (days)	14.5(7.0-25.8) (n=50)	10.0 (7.0-15.0) (n=165)	0.018
Symptoms			
Fever	48 (72.7)	174 (79.5)	0.248
Expectoration	13 (25.0)	43 (26.1)	0.879
Fatigue	15 (28.8)	64 (38.8)	0.194
Myalgia	8 (15.4)	32 (19.4)	0.516
Headache	5 (5.8)	9 (5.5)	1.000*
Pharyngalgia	2 (3.8)	4 (2.4)	0.631*
Rhinorrhea	1 (1.9)	10 (6.1)	0.467*
Pectoralgia	3 (5.8)	7 (4.2)	0.706*
Diarrhea	4 (7.7)	24 (14.5)	0.242*
Nausea	2 (3.8)	13 (7.9)	0.531*
Vomiting	1 (1.9)	9 (5.5)	0.458*
Signs			
Temperature	36.6 (36.5-37.0) (n=48)	36.7 (36.3-37.2) (n=156)	0.839
MAP (mmHg)	99.67 (91.58-106.67) (n=40)	96.67 (91.00-105.75) (n=146)	0.325
Heart rate (/min)	85.5 (77.0-101.8) (n=48)	85.0 (76.0-98.0) (n=158)	0.186
Respiratory rate (/min)	20.0 (20.0-20.8) (n=48)	20.0 (20.0-24.0) (n=151)	0.080
Laboratory findings			
WBC (×10^9^/L)	5.40 (4.31-6.67)	6.07 (4.66-8.39)	0.053
RBC (×10^9^/L)	3.95±0.54 (n=52)	4.01±0.59 (n=164)	0.516
Platelets (×10^9^/L)	195.5 (161.3-258.0)	213.0 (159.5-268.5)	0.585
Neutrophils (×10^9^/L)	3.53 (2.61-4.74) (n=52)	4.25 (3.16-6.78) (n=164)	0.005
Lymphocytes (×10^9^/L)	1.16 (0.90-1.56)	0.88 (0.62-1.19)	<0.001
Monocytes (×10^9^/L)	0.41 (0.32-0.48) (n=52)	0.40 (0.30-0.57) (n=162)	0.821
AST (U/L)	29.5 (22.3-39.5)	31.0 (23.0-43.0)	0.199
ALT (U/L)	25.0 (16.0-45.8)	30.0 (21.0-47.5)	0.108
ALP (U/L)	63.5 (48.5-74.8)	59.0 (47.0-77.0)	0.628
LDH (U/L)	218.0 (178.0-278.5)	299.0 (210.5-378.5)	<0.001
GGT (U/L)	25.5 (17.3-56.5)	29.0 (19.0-45.5)	0.512
TBIL (μmol/L)	10.25 (7.35-13.10)	11.80 (8.80-15.70)	0.068
DBIL(μmol/L)	3.00 (2.35-4.18)	3.80 (2.75-5.30)	0.010
IBIL (μmol/L)	7.10 (5.60-9.70) (n=51)	8.00 (5.60-10.70) (n=163)	0.454
Total protein (g/L)	64.51±6.62	62.34±6.20	0.032
ALB (g/L)	33.71±4.81	29.25±4.93	<0.001
Globulin (g/L)	31.34±4.56 (n=50)	33.04±5.18 (n=165)	0.039
TBA (μmol/L)	4.20 (2.80-6.29) (n=52)	2.70 (1.80-4.40) (n=163)	0.004
BUN (mmol/L)	4.88 (3.73-5.90) (n=52)	5.26 (4.06-7.44) (n=161)	0.063
Creatinine (μmol/L)	67.95 (57.28-80.80) (n=52)	71.55 (59.55-88.30) (n=162)	0.445
Uric acid (μmol/L)	260.65 (205.80-322.63) (n=52)	242.95 (186.65-296.48) (n=162)	0.216
Glucose (mmol/L)	5.89 (5.26-7.37) (n=52)	6.40 (5.51-8.03) (n=160)	0.099
CK (U/L)	80.0 (53.0-125.0) (n=39)	74.0 (46.0-126.0) (n=131)	0.370
CK-MB (U/L)	12.0 (8.0-15.0) (n=39)	11.0 (9.0-15.0) (n=131)	0.927
CRP (mg/L)	6.18 (1.65-33.84) (n=46)	33.73 (11.23-71.34) (n=155)	<0.001
D-dimer (μg/L)	0.58 (0.29-1.30) (n=44)	1.15 (0.41-4.46) (n=147)	0.004
PT (s)	12.70 (12.20-13.40) (n=52)	13.50 (12.80-14.68) (n=164)	<0.001
APTT (s)	34.90 (32.90-39.10) (n=52)	37.30 (33.70-41.68) (n=164)	0.046
Fibrinogen (g/L)	3.62 (3.06-4.66)	4.53 (3.51-5.19)	0.016
Thrombin time (s)	15.40 (14.93-16.40)	15.80 (15.00-16.70)	0.097
Procalcitonin (ng/mL)	0.05 (0.04-0.09) (n=39)	0.11 (0.06-0.25) (n=113)	<0.001
NLR	2.90 (1.89-4.63) (n=52)	4.92 (2.97-10.00) (n=164)	<0.001
PLR	161.59 (124.90-245.59)	248.53 (172.48-335.71)	<0.001
LMR	2.88 (2.16-3.92) (n=52)	2.27 (1.48-3.18) (n=162)	<0.001
SII	589.49 (354.76-1152.13) (n=52)	1067.33 (626.84-1948.19) (n=164)	<0.001
ANRI	8.81 (5.16-12.09) (n=52)	7.02 (4.31-11.39) (n=164)	0.287
APRI	0.37 (0.26-0.51)	0.38 (0.26-0.65)	0.429
ALRI	24.36 (16.24-36.21)	36.26 (22.55-60.95)	<0.001
LCR	0.19 (0.03-0.94) (n=46)	0.03 (0.01-0.10) (n=155)	<0.001

OH, onset-to-hospitalization; MAP, mean arterial pressure; WBC, white blood cell; RBC, red blood cell; AST, aspartate aminotransferase; ALT, Alanine transaminase; ALP, alkaline phosphatase; LDH, lactic dehydrogenase; GGT, gamma-glutamyl transpeptidase; TBIL, total bilirubin; DBIL, direct bilirubin; IBIL, indirect bilirubin; ALB: albumin; TBA, total bile acid; BUN, blood urea nitrogen; CK: Creatine kinase; CK-MB: Creatine kinase-MB; CRP, C-reactive protein; PT: prothrombin time; APTT, activated partial thromboplastin time; NLR, neutrophil-to-lymphocyte ratio; PLR, platelet-to-lymphocyte ratio; LMR, lymphocyte-to-monocyte ratio; SII, systemic Immune-inflammation index; ANRI, AST-to-neutrophil ratio index; APRI, AST-to-platelet ratio index; ALRI, AST-to-lymphocyte ratio index; LCR, lymphocyte-to-CRP ratio.

* Calculated with Fisher’s exact test.

### Identification of significant predictors for severity of elderly COVID-19 patients

**Figure 2:**
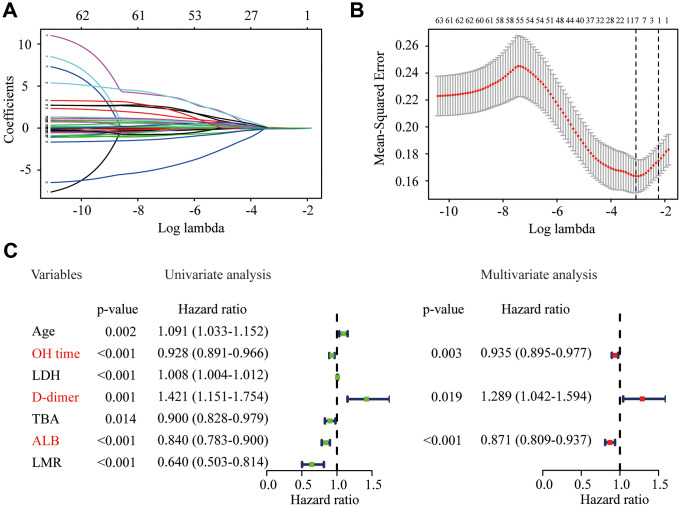
**Identification of significant predictors for the severity of COVID-19 in elderly patients.** (**A**) LASSO coefficient profiles of the candidate predictors. (**B**) Selection of the optimal penalization coefficient in the LASSO regression. (**C**) Univariate and multivariate logistic regression of the predictors.

A total of 63 potential predictors from 217 elderly COVID-19 patients in the training cohort were enrolled in LASSO regression, and seven candidate predictors were selected, including age, OH time, lactic dehydrogenase (LDH), d-dimer, total bile acid (TBA), ALB and lymphocyte-to-monocyte ratio (LMR) (Figure 2A and 2B). These predictors were then entered into the univariate logistic regression, and all the predictors were significantly correlated with the severity of COVID-19 (Figure 2C). Furthermore, we recruited all these predictors in multivariate logistic regression to adjust the effects of covariates for the presence of severe or critical COVID-19. The results demonstrated that OH time (odds ratio [OR] = 0.935, 95% confidence interval [CI]: 0.895 - 0.977, P = 0.003), d-dimer (OR = 1.289, 95% CI: 1.042 - 1.594, P = 0.019) and ALB (OR = 0.871, 95% CI: 0.809 - 0.937, P < 0.001) were significant predictors for the severity of COVID-19 in elderly patients (Figure 2C). Then, we used logistic regression, decision tree, and support vector machine (SVM) to construct the different predictive models. The receiver operating characteristic (ROC) curve indicated that logistic regression was as good as the SVM and better than the decision tree (Supplementary Figure 1). Therefore, logistic regression model was used for further analysis due to its better performance.

### Nomogram development for severity prediction in elderly COVID-19 patients

**Figure 3:**
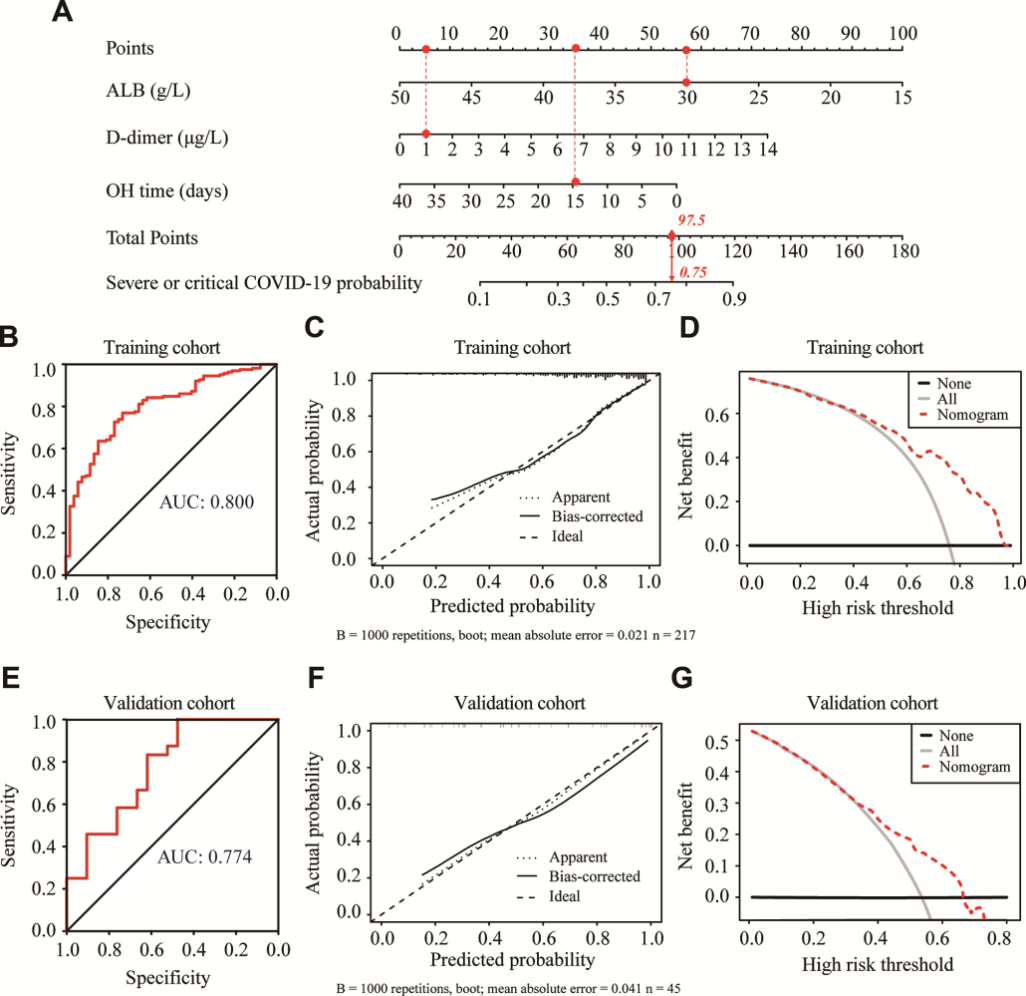
**Construction and validation of the predictive nomogram for the severity of COVID-19 in elderly patients.** (**A**) Development of the nomogram to predict the severity of COVID-19 in elderly patients. For example, if the albumin (ALB), d-dimer and onset to hospitalization (OH) time of an admitted elderly COVID-19 patient were 30 g/L, 1 μg/L and 15 days, respectively, the corresponding points for ALB, d-dimer and OH time were 57.5, 5 and 35, respectively. The total points value for this patient was 97.5, with a probability of 0.75 for developing severe or critical illness after admission. (**B**, **E**) Receiver operating characteristic (ROC) curves of the nomogram in the training cohort (**B**) and validation cohort (**E**). (**C**, **F**) Calibration curve of the nomogram in the training cohort (**C**) and validation cohort (**F**). (**D**, **G**) Decision curve analysis in the training cohort (**D**) and validation cohort (**G**). The y-axis represents net benefits, calculated by subtracting the relative harms (false positives) from the benefits (true positives). The x-axis measures the threshold probability.

Based on the significant predictors, we established a predictive nomogram for the severity of COVID-19 in elderly patients (Figure 3A). These three predictors were assigned a score ranging from 0 to 100 on a point scale. The probability of severe or critical COVID-19 could be efficiently estimated by calculating the total score of these three predictors and placing the total score on a total point scale. The sensitivity and specificity for predicting the severity of COVID-19 at different cutoff values are summarized in Table 3. Based on the maximum Youden index, the optimal cutoff value of the nomogram-predicted probability was set as 0.722. At this cutoff, the sensitivity, specificity, positive predictive value, and negative predictive value, when used in differentiating the presence from absence of severe or critical COVID-19, were 77.0%, 73.1%, 90.1%, and 50.0%, respectively (Table 4).

**Table 3:** Differential efficacy of the nomogram at different predicted probability.

**Predicted probability**	**Sensitivity**	**Specificity**	**PPV**	**NPV**
Training cohort				
0.50	95.8%	25.0%	80.2%	65.0%
0.60	90.9%	38.5%	82.4%	57.1%
0.70	80.6%	65.4%	88.1%	51.5%
0.80	61.2%	84.6%	92.7%	40.7%
Validation cohort				
0.5	100.0%	23.8%	60.0%	100.0%
0.6	100.0%	38.1%	64.9%	100.0%
0.7	79.2%	47.6%	63.3%	66.7%
0.8	50.0%	71.4%	66.7%	55.6%

PPV, positive predictive value; NPV, negative predictive value.

**Table 4:** Differential efficacy of the nomogram at optimal predicted probability.

**Variables**	**Value**
Sensitivity	77.0%
Specificity	73.1%
Positive predictive value	90.1%
Negative predictive value	50.0%
Positive likelihood ratio	2.86
Negative likelihood ratio	0.31
ROC area (95%CI)	0.800 (0.734-0.866)
Predicted probability	0.722

CI, confidence intervals; ROC, receiver operating characteristic.

### Validation of the predictive nomogram

In the training cohort, the nomogram showed good discrimination for predicting the severity of COVID-19, with the C index of 0.800 (Figure 3B). The calibration curve graphically showed good agreement between the predicted and actual severity classifications of COVID- 19 (Figure 3C). Decision curve analysis (DCA) was used to evaluate the clinical utility of the nomogram by quantifying the probabilities of net benefits at a threshold from 0.0 to 1.0. DCA results showed that using this nomogram to predict the severity of COVID-19 had more benefits than the measures that treat all patients or treat none of patients (Figure 3D). In the validation cohort, the C index for the nomogram was 0.774 (Figure 3E). The calibration curve graphically showed good agreement between the predicted and observed curves (Figure 3F). DCA results further validated the clinical usefulness of our nomogram (Figure 3G).

## DISCUSSION

COVID-19 has spread globally rapidly, and no specific medication for COVID-19 has been identified [16]. Some models have been proposed to predict the severity of COVID-19, most of which mainly focus on general patients [10, 17–20], and little attention has been paid specifically to elderly patients. Considering that elderly patients were more likely to progress to severe or critical COVID-19 [6], early identification of elderly COVID-19 patients at high risk of progression to severe or critical pneumonia will facilitate better use of medical resources and early appropriate supportive care.

In our study, ALB, OH time and d-dimer were finally selected as predictors for the severity of COVID-19 in elderly patients. Based on these predictors, a risk nomogram with the C index of 0.800 was established for the prediction of severe or critical COVID-19, suggesting that our nomogram had good discrimination. The calibration curve demonstrated excellent consistency between the prediction of our nomogram and the observed curve. DCA further showed that our nomogram conferred significantly high clinical net benefits. More importantly, our nomogram worked well in an external validation cohort. These findings suggested that our nomogram was of significant value for accurate individual assessment of the incidence of severe or critical COVID-19 in elderly patients.

ALB has been reported to be negatively correlated with the severity of COVID-19 [10]. As previously stated, inflammatory cells were found in the hepatic sinuses from a dead COVID-19 patient [21], suggesting that SARS-CoV-2 could cause damage to the liver. Considering that ALB is synthesized in the liver, decreased ALB levels indirectly reflect the extent of liver injury [22]. Usually, severe or critical COVID-19 patients are more likely to have liver injury than mild or moderate COVID-19 patients [23]. This might be the reason why patients in the severe group had decreased ALB levels compared with those in the non-severe group. Moreover, we found that OH time was shorter in the severe group than in the non-severe group and negatively associated with the severity of COVID-19. Chen and his team arrived at a similar conclusion [24]. Chen et al. compared the clinical characteristics between older patients and younger patients with COVID-19 and found that older patients have shorter OH times. Additionally, by analyzing survival and non-survival patients, they found that non-survival patients had shorter OH times than those who survived [24]. Previous studies also demonstrated that patients with progression often exhibited exacerbation within one week of disease onset [25, 26]. The detailed mechanism is still unknown and needs further investigation. For d-dimer, we found a higher level of d-dimer in the severe group than in the non-severe group, and d-dimer was positively related to the severity of COVID-19. A previous study conducted by Roselo et al. demonstrated that d-dimer was a significant prognostic factor in patients with infection and sepsis [27]. Regarding the role of d-dimer in COVID-19 patients, Zhou and his team found that a d-dimer level greater than 1 μg/mL was associated with the death of COVID-19 patients [28]. Zhang et al. reached a similar conclusion, stating that a d-dimer level greater than 2.0 μg/mL on admission could effectively predict in-hospital mortality in COVID-19 patients [29]. Collectively, d-dimer could act as an early and helpful marker of the severity ofCOVID-19.

Numerous studies have shown that patients with comorbidities are more likely to develop severe or critical pneumonia [30, 31]. Our study did not find significant differences in tumor, diabetes, hypertension, coronary heart disease, chronic kidney disease or chronic respiratory disease between the severe and non-severe groups. Additionally, age has been reported to be correlated with the severity of COVID-19 [32, 33]. However, in our study, age was not included after LASSO regression, which suggested that other parameters had better predictive abilities than age for COVID-19 severity prediction in elderly patients.

To our knowledge, this is the first nomogram for predicting the incidence of severe or critical COVID-19 in elderly patients. The greatest strength is that this practical quantitative prediction tool is inexpensive and easily used and popularized because only three parameters are needed, which are easily accessible in clinical practice. However, we must admit that our study has some limitations. First, this was a retrospective study including only 262 elderly COVID-19 patients. Larger prospective studies are needed to validate the findings. Second, this nomogram was constructed and validated based on data from China. External validation from other countries and races is necessary to confirm the predictive value of the nomogram. Third, our model was trained on patients who were not randomized or matched, and we did not approach the problem of unbalanced data between severe and non-severe patients. Finally, due to the analysis being based on clinicopathologic and laboratory data, we did not include specific markers such as IgM and IgG antibody detection, which might further improve the accuracy of the nomogram.

In conclusion, we demonstrated that ALB, d-dimer and OH time are significant predictors of the severity of COVID-19 in elderly patients. By combining these easily accessible predictive factors, a risk nomogram was established to predict the incidence of severe or critical COVID-19 in elderly patients. The nomogram could optimally assist in alleviating medical resources limitations and reducing poor outcomes.

## MATERIALS AND METHODS

### Data collection

The training cohort data were collected on admission, including demographic, clinical and laboratory characteristics of laboratory-confirmed cases of COVID-19 from Jan 25 to Mar 14, 2020, at the Union Hospital of Huazhong University of Science and Technology. Patients less than 60 years old were excluded, and 217 elderly patients (age ≥ 60 years) with COVID-19 were included in the study. The validation cohort data of 45 elderly COVID-19 patients were retrospectively collected from five hospitals (Harbin Chest Hospital, Harbin Infectious Disease Hospital, Jilin Infectious Disease Hospital, Harbin Second Hospital, and Heilongjiang Provincial Hospital for Prevention and Treatment of Infectious Diseases) between April 8 and May 11, 2020. None of the patients enrolled in the two cohorts were randomized or matched. All patients were SARS-CoV-2 RNA positive and could be divided into a severe group or a non-severe group. In the severe group, patients progressed to severe or critical COVID-19 during hospitalization; in the non-severe group, patients maintained non-severe conditions (recovery or mild or moderate COVID-19) during hospitalization. This study was approved by the ethics committee of each hospital for emerging infectious diseases. The ethics committee of the hospital waived written informed consent from patients with COVID-19.

The diagnosis and severity classification of COVID-19 were based on the New Coronavirus Pneumonia Prevention and Control Program published by the National Health Commission of China [34]. Mild pneumonia indicates asymptomatic infection or mild clinical symptoms without abnormal chest imaging findings. Moderate pneumonia indicates the presence of both clinical symptoms and abnormal chest imaging findings. Patients are diagnosed with severe pneumonia when the disease progresses to meet any of the following conditions: (1) significantly increased respiration rate: RR ≥ 30/min; (2) oxygen saturation ≤ 93% in the rest state; and (3) PaO2/FiO2 ≤ 300 mmHg (1 mmHg = 0.133 kPa). Critical pneumonia occurs when the disease progresses rapidly with any of the following conditions: (1) respiratory failure, which requires mechanical ventilation; (2) shock; and (3) other organ failures needing monitoring and treatment in the ICU.

### Clinicopathologic variables

Patients’ basic information was obtained, including age, gender, comorbidities, OH time, symptoms (including fever, expectoration, fatigue, myalgia, headache, pharyngalgia, rhinorrhea, pectoralgia, diarrhea, nausea and vomiting) and signs (including body temperature, mean arterial pressure, heart rate and respiratory rate). The laboratory parameters measured included white blood cells, red blood cells, platelets, neutrophils, lymphocytes, monocytes, aspartate aminotransferase (AST), alanine transaminase, alkaline phosphatase, LDH, gamma-glutamyl transpeptidase, total bilirubin, direct bilirubin, indirect bilirubin, total protein, ALB, globulin, TBA, blood urea nitrogen, creatinine, uric acid, blood glucose, creatine kinase, creatine kinase-MB, C-reactive protein (CRP), d-dimer, prothrombin time, activated partial thromboplastin time, fibrinogen, thrombin time and procalcitonin. In addition, some inflammatory markers were calculated from the admission full blood counts, AST and CRP, including the neutrophil-lymphocyte ratio, platelet-lymphocyte ratio, lymphocyte-to-monocyte ratio, systemic immune-inflammation index, AST-to-neutrophil ratio index, AST-to-platelet ratio index, AST-to-lymphocyte ratio index and lymphocyte-to-CRP ratio.

### Statistical analysis

Continuous variables were expressed as the mean ± standard deviation or median (IQR) for normal or non normal distributions, respectively, followed by an unpaired t-test or Wilcoxon rank sum test. Categorical variables were summarized as counts (percentages) and compared using the chi-square test or Fisher’s exact test, as appropriate. P < 0.05 was considered statistically significant. SPSS 22.0 (SPSS Inc, Chicago, IL, USA) software was used to analyze the above data.

Considering that all the potential predictors had 2.4% missing values in the training cohort, we adopted multiple imputation using the “mice” package in R software (R version 3.6.3) to impute the missing values. After multiple imputation, the OR and P value were unchanged in all of these potential predictors except procalcitonin, which was excluded in our subsequent analysis (Supplementary Table 1). To generate sparse coefficients that allow us to select features for prediction, we used logistic regression with L1 regularization (LASSO) to select the significant predictors based on the Akaike information criteria. Cross-validation was used to estimate LASSO hyper-parameters. Furthermore, we constructed predictive models using logistic regression, decision tree and SVM using the R packages “rpart”, “rpart.plot” and “e1071”. Finally, by combining these significant predictors, we established a nomogram to predict the incidence of severe or critical COVID-19 for elderly patients on admission. ROC curves and calibration curves were plotted to assess the discrimination and accuracy of the nomogram. Bootstrapping aggregating method was used to obtain the estimates in the calibration of models. All data in the training set with 1000 repetitions of resampling were applied in the bootstrapping. DCA was conducted to evaluate the clinical utility of the nomogram by quantifying net benefits against a range of threshold probabilities [35, 36]. For external validation of the nomogram, the established nomogram was used to calculate the total points of each patient in the validation cohort. The ROC curve, calibration curve and DCA results were plotted to externally evaluate the application scope of the nomogram. The R packages “rms”, “pROC” and “dca.R” were used in these analyses.

### Ethics committee approval

Reviewed and approved by the institutional research ethics boards of the designated hospitals in our study and Xiangya Hospital, Central South University (Changsha, China); approval number: 202002024.

## Supplementary Material

Supplementary Figure 1

Supplementary Table 1
