# Supplementary material for: A predictive model for the severity of COVID-19 in elderly patients
Source: Aging (Albany NY). 2020 Nov 10;12(21):20982–96. doi: 10.18632/aging.103980 (PMC7695402; doi:10.18632/aging.103980)
Supplement: Supplementary Figure 1 [file aging-12-103980-s001..pdf]

SUPPLEMENTARY FIGURE

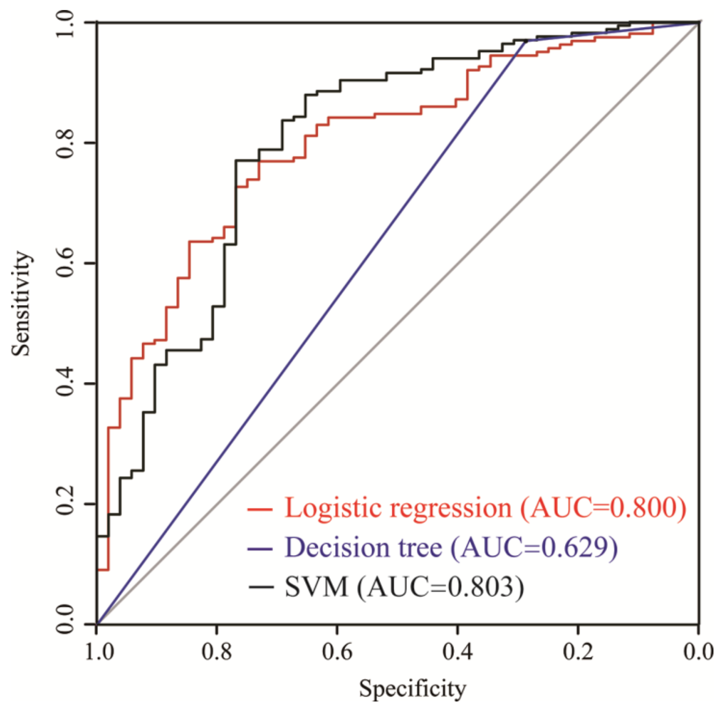

Supplementary Figure 1. Receiver operating characteristic (ROC) curves of different predictive models using logistic regression (red), decision tree (blue), and support vector machine (black).
