# Supplementary material for: A predictive model for the severity of COVID-19 in elderly patients
Source: Aging (Albany NY). 2020 Nov 10;12(21):20982–96. doi: 10.18632/aging.103980 (PMC7695402; doi:10.18632/aging.103980)
Supplement: Supplementary Table 1 [file aging-12-103980-s002..pdf]

SUPPLEMENTARY TABLE

Supplementary Table 1. Association of the clinical characteristics with severity in elderly COVID-19 patients.

Variables	Analysis with observed data, β (95% CI)	p-value	Analysis with multiple imputation data, β (95% CI)	p-value
Age (year)	1.091 (1.033-1.152)	0.002	1.091(1.033-1.152)	0.002
Gender, female vs. male	0.554 (0.295-1.041)	0.066	0.554 (0.295-1.041)	0.066
Comorbidities, yes vs. no	1.762 (0.934-3.324)	0.080	1.762 (0.934-3.324)	0.080
OH time (days)	0.928 (0.890-0.966)	<0.001	0.928 (0.891-0.966)	<0.001
Symptoms, yes vs. no				
Fever	1.752 (0.856-3.585)	0.125	1.752 (0.856-3.585)	0.125
Expectoration	1.057 (0.516-2.167)	0.879	1.057 (0.516-2.167)	0.879
Dyspnea	1.959 (1.025-3.744)	0.042	1.959 (1.025-3.744)	0.042
Fatigue	1.563 (0.794-3.075)	0.196	1.563 (0.794-3.075)	0.196
Myalgia	1.323 (0.568-3.085)	0.517	1.323 (0.568-3.085)	0.517
Headache	0.942 (0.245-3.619)	0.931	0.942 (0.245-3.619)	0.931
Pharyngalgia	0.612 (0.110-3.492)	0.589	0.612 (0.110-3.492)	0.589
Rhinorrhea	3.290 (0.411-26.333)	0.262	3.290 (0.411-26.333)	0.262
Pectoralgia	0.724 (0.180-2.905)	0.648	0.724 (0.180-2.905)	0.648
Diarrhea	2.043 (0.674-6.186)	0.206	2.043 (0.674-6.186)	0.206
Nausea	2.138 (0.466-9.802)	0.328	2.138 (0.466-9.802)	0.328
Vomiting	2.942 (0.364-23.789)	0.312	2.942 (0.364-23.789)	0.312
Signs				
MAP (mmHg)	0.987 (0.957-1.019)	0.420	0.990 (0.962-1.109)	0.487
Heart rate (/min)	0.986 (0.966-1.005)	0.154	0.987 (0.968-1.006)	0.186
Respiratory rate (/min)	1.105 (1.004-1.216)	0.041	1.074 (0.987-1.168)	0.096
Temperature (°C)	0.966 (0.611-1.528)	0.882	0.929 (0.594-1.405)	0.749
Laboratory findings				
WBC ($\times 10^9$/L)	1.128 (0.997-1.277)	0.056	1.128 (0.997-1.277)	0.056
RBC ($\times 10^9$/L)	1.199 (0.695-2.070)	0.514	1.180 (0.685-2.034)	0.550
Platelets ($\times 10^9$/L)	1.000 (0.997-1.004)	0.870	1.000 (0.997-1.004)	0.870
Neutrophils ($\times 10^9$/L)	1.185 (1.031-1.362)	0.017	1.189 (1.034-1.366)	0.005
Lymphocytes ($\times 10^9$/L)	0.312 (0.164-0.596)	<0.001	0.312 (0.164-0.596)	<0.001
Monocytes ($\times 10^9$/L)	2.742 (0.520-14.467)	0.235	2.844 (0.536-15.089)	0.220
AST (U/L)	1.012 (0.995-1.029)	0.158	1.012 (0.995-1.029)	0.158
ALT (U/L)	1.004 (0.994-1.013)	0.487	1.004 (0.994-1.013)	0.487
ALP (U/L)	1.000 (0.990-1.010)	0.982	1.000 (0.990-1.010)	0.982
LDH (U/L)	1.008 (1.004-1.012)	<0.001	1.008 (1.004-1.012)	<0.001
GGT (U/L)	1.001 (0.996-1.007)	0.602	1.001 (0.996-1.007)	0.602
TBIL (μmol/L)	1.059 (0.992-1.132)	0.087	1.059 (0.992-1.132)	0.087
DBIL(μmol/L)	1.253 (1.030-1.524)	0.024	1.253 (1.030-1.524)	0.024
IBIL (μmol/L)	0.990 (0.953-1.029)	0.617	0.993 (0.955-1.032)	0.714

Total protein (g/L)	0.948 (0.903-0.996)	0.034	0.948 (0.903-0.996)	0.034
ALB (g/L)	0.840 (0.783-0.900)	<0.001	0.840 (0.783-0.900)	<0.001
Globulin (g/L)	1.074 (1.003-1.151)	0.040	1.084 (1.013-1.161)	0.019
TBA (μmol/L)	0.902 (0.829-0.980)	0.015	0.900 (0.828-0.979)	0.014
BUN (mmol/L)	0.989 (0.952-1.027)	0.561	0.988 (0.951-1.027)	0.541
Creatinine (μmol/L)	0.999 (0.998-1.001)	0.448	0.999 (0.998-1.001)	0.436
Uric acid (μmol/L)	0.999 (0.996-1.001)	0.335	0.999 (0.996-1.001)	0.314
Glucose (mmol/L)	1.100 (0.972-1.244)	0.130	1.102 (0.974-1.246)	0.122
CK (U/L)	1.001 (0.998-1.004)	0.504	1.001 (0.999-1.003)	0.308
CK-MB (U/L)	1.006 (0.970-1.044)	0.743	1.015 (0.984-1.047)	0.354
CRP (mg/L)	1.020 (1.008-1.031)	0.001	1.018 (1.007-1.029)	0.001
D-dimer (μg/L)	1.346 (1.096-1.653)	0.005	1.421 (1.151-1.754)	0.001
PT (s)	2.091 (1.475-2.966)	<0.001	2.099 (1.482-2.972)	<0.001
APTT (s)	1.042 (0.993-1.094)	0.091	1.042 (0.993-1.094)	0.092
Fibrinogen (g/L)	1.262 (0.980-1.626)	0.072	1.262 (0.980-1.626)	0.072
Thrombin time (s)	1.291 (0.998-1.668)	0.052	1.291 (0.998-1.668)	0.052
NLR	1.125 (1.032-1.227)	0.007	1.127 (1.034-1.229)	0.007
PLR	1.004 (1.001-1.007)	0.005	1.004 (1.001-1.007)	0.005
LMR	0.638 (0.501-0.812)	<0.001	0.640 (0.503-0.814)	<0.001
SII	1.000 (1.000-1.001)	0.011	1.000 (1.000-1.001)	0.010
ANRI	0.993 (0.953-1.034)	0.739	0.992 (0.953-1.034)	0.716
APRI	1.840 (0.740-4.576)	0.189	1.840 (0.740-4.576)	0.189
ALRI	1.019 (1.006-1.033)	0.005	1.019 (1.006-1.033)	0.005
LCR	0.922 (0.855-0.994)	0.034	0.926 (0.859-0.997)	0.042

OH, onset-to-hospitalization; MAP, mean arterial pressure; WBC, white blood cell; RBC, red blood cell; AST, aspartate aminotransferase; ALT, Alanine transaminase; ALP, alkaline phosphatase; LDH, lactic dehydrogenase; GGT, gamma-glutamyl transpeptidase; TBIL, total bilirubin; DBIL, direct bilirubin; IBIL, indirect bilirubin; ALB: albumin; TBA, total bile acid; BUN, blood urea nitrogen; CK: Creatine kinase; CK-MB: Creatine kinase-MB; CRP, C-reactive protein; PT: prothrombin time; APTT, activated partial thromboplastin time; NLR, neutrophil-to-lymphocyte ratio; PLR, platelet-to-lymphocyte ratio; LMR, lymphocyte-to-monocyte ratio; SII, systemic Immune-inflammation index; ANRI, AST-to-neutrophil ratio index; APRI, AST-to-platelet ratio index; ALRI, AST-to-lymphocyte ratio index; LCR, lymphocyte-to-CRP ratio.
